# Selection of Bacterial Mutants in Late Infections: When Vector Transmission Trades Off against Growth Advantage in Stationary Phase

**DOI:** 10.1128/mBio.01437-19

**Published:** 2019-10-08

**Authors:** Marine C. Cambon, Nathalie Parthuisot, Sylvie Pagès, Anne Lanois, Alain Givaudan, Jean-Baptiste Ferdy

**Affiliations:** a Évolution et Diversité Biologique, CNRS–Université Paul Sabatier, Toulouse, France; b Diversité, Génome et Interactions Microorganismes Insectes, INRA–Université Montpellier, Montpellier, France; University of Hawaii at Manoa

**Keywords:** *Xenorhabdus nematophila*, GASP, transmission, within-host evolution

## Abstract

Bacterial infections are often composed of cells with distinct phenotypes that can be produced by genetic or epigenetic mechanisms. This phenotypic heterogeneity has proved to be important in many pathogens, because it can alter both pathogenicity and transmission. We studied how and why it can emerge during infection in the bacterium Xenorhabdus nematophila, a pathogen that kills insects and multiplies in the cadaver before being transmitted by the soil nematode vector Steinernema carpocapsae. We found that phenotypic variants cluster in three groups, one of which is composed of *lrp* defective mutants. These mutants, together with variants of another group, have in common that they maintain high survival during late stationary phase. This probably explains why they increase in frequency: variants of X. nematophila with a growth advantage in stationary phase (GASP) are under strong positive selection both in prolonged culture and in late infections. We also found that the within-host advantage of these variants seems to trade off against transmission by nematode vectors: the variants that reach the highest load in insects are those that are the least transmitted.

## Abstract (precis)

Pathogens can evolve inside their host, and the importance of this mutation-fueled process is increasingly recognized. A disease outcome may indeed depend in part on pathogen adaptations that emerge during infection. It is therefore important to document these adaptations and the conditions that drive them. In our study, we took advantage of the possibility to monitor within-host evolution in the insect pathogen *X. nematophila*. We demonstrated that selection occurring in aged infection favors *lrp* defective mutants, because these metabolic mutants benefit from a growth advantage in stationary phase (GASP). We also demonstrated that these mutants have reduced virulence and impaired transmission, modifying the infection outcome. Beyond the specific case of *X. nematophila*, we propose that metabolic mutants are to be found in other bacterial pathogens that stay for many generations inside their host.

## INTRODUCTION

Bacterial infections, even when they started from a single pathogenic strain, often end up being composed of cells with different phenotypes (1). In cases where groups of cells with distinct phenotypes interact to better exploit their host (2), the molecular mechanisms driving bacterial diversity can be considered adaptations, which ultimately increase pathogen transmission. To demonstrate this theory, it is necessary to understand both what these mechanisms are and how they impact the success of the infection.

Mutation is probably the most obvious mechanism that produces diversity during infection. Its importance is increasingly admitted (1), notably because cases are now accumulating where it fuels the evolution of pathogens inside the host, in particular in human diseases (3, 4). While mutation generally occurs throughout the whole genome, there are other mechanisms that impact a restricted set of genes. These mechanisms can be epigenetic alterations, where clonal populations of bacteria modify their phenotype by changing their regulatory state (5, 6). These can also be genetic alterations, as in phase variation where a specific high-rate mutation mechanism produces reversible changes in one or a few genes (7, 8). Phase variation is described as the basic mechanism that makes antigenic variation a successful instrument for some pathogens to escape their host immune system (9).

The insect bacterial pathogen *Xenorhabdus* is a promising model to study the adaptive nature of mechanisms that control phenotypic variation inside an infection. This pathogen kills insects and proliferates in their cadavers for 1 or 2 weeks, before it is transmitted by the nematode vector *Steinernema* (10). During this long period, *Xenorhabdus* maintains high densities inside the insect and, therefore, potentially accumulates phenotypic variation. As the complete life cycle of *Xenorhabdus* can be experimentally reproduced (11), it is possible to quantify how this variation impacts each of its different stages.

*Xenorhabdus* isolated from the wild typically are in a form described as primary, but in culture media, they convert to another, secondary form when reaching long-term stationary phase (12). In his seminal paper, Akhurst (13) showed that secondary forms of *Xenorhabdus* also appear during infection and that this occurs at a rate that varies greatly among *Xenorhabdus* strains. Although the phenotypic differences between the two forms can also vary depending on strain and species, only cells of the primary form are able to bind bromothymol blue dye, are motile, agglutinate red blood cells, and produce fimbriae, hemolysins, proteases, antimicrobials, and crystalline inclusion bodies (12, 14–17). Interestingly, genes for which expression differs between primary and secondary forms have also been shown to play a role in the interaction between Xenorhabdus nematophila, its nematode vector, and its insect target (18). This strongly suggests that the emergence of secondary forms should impact X. nematophila interactions with their invertebrate hosts. This could also explain why nematodes have so far been reported to carry primary forms, although previous experiments suggest that secondary forms of *X. nematophila* are capable of both killing insects (16) and being transmitted by the nematode Steinernema carpocapsae (19).

The alternation between primary and secondary forms has so far been interpreted as a case of phase variation (12), and later termed phenotypic variation (17). As the phenotype of secondary forms matches that of *lrp* defective mutants (20–24), it has also been proposed that the Lrp master regulator might be involved in the production of secondary variants in *X. nematophila* (20). However, the mechanism driving phenotypic variation in *X. nematophila* is unknown.

The goals of this study were to better understand (i) the molecular mechanisms that are responsible for the production of phenotypic variants and (ii) the impact of these variants on transmission by the vector. To do so, we investigated a large collection of *X. nematophila* isolates with various phenotypic forms. We found that secondary forms are not phase variants but rather plain *lrp* mutants. We also found that at least a third phenotypic form exists in *X. nematophila* which is not a *lrp* mutant. All these variants have a growth advantage in stationary phase (GASP [25]) which probably explains why they reach higher loads than primary forms during late infection. We then quantified how these variants are transmitted by the nematode vector S. carpocapsae and found indications that isolates that reach the highest densities in insects are the least transmitted by nematodes. We therefore propose that *X. nematophila* experiences a trade-off between traits that are favored during late infection and traits that increase transmission.

## RESULTS

### Variant isolates of *X. nematophila* can be classified into three phenotypic groups.

**FIG 1:**
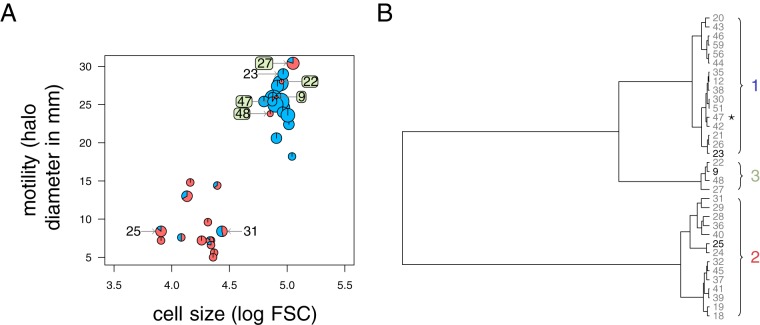
Variants of *X. nematophila* can be classified in three phenotypic groups. (A) Characterization of the 34 isolates. For each isolate, average log forward scatter (FSC), a proxy for cell size, and average motility halo diameter, a measure of cell motility, are represented. The red part of the small pie charts indicates the proportion of red colonies observed for each isolate on NBTA culture medium, and the pie chart diameter increases with average measure of antibiotic activity. The boxed numbers identify the five isolates that constitute the third group. Other numbers identify isolates that we will use most often as representative of their group (G1#23 and G2#25 for group 1 and 2, respectively) or that we discuss specifically in text (G2#31). (B) Result of a hierarchical clustering analysis (using a unweighted-pair group method using average linkages [UPGMA] method) based on log FSC, motility, and proportion of red colonies. The asterisk indicates variant #47, which we added to group 3 but clusters with group 1. Boldface numbers identify isolates that we will use most often as representative of their group.

Variant isolates of *X. nematophila* are generally classified in two groups. Compared to group 1, group 2 variants cannot adsorb dye, are not motile, do not secrete antibiotics, and have weak or no hemolytic and lipolytic activities (13). Among our 34 isolates, 14 had such characteristics (Fig. 1A and Table 1). Remarkably, these group 2 variants also had smaller cells than those of group 1 (Fig. 1A), which adds a new characteristic to their set of phenotypes.

**TABLE 1:** Average phenotypic characteristics (± standard error) of the three variant groups^*a*^

Phenotypic characteristic	Value for parameter (mean ± SE)		
	Group 1 (*n* = 15)	Group 2 (*n* = 14)	Group 3 (*n* = 5)
Dye adsorption	Yes	No	No
Motility (halo [mm])	24.72 ± 0.71	8.73 ± 0.84	26.72 ± 1.14
Cell size (FSC)	4.94 ± 0.02	4.24 ± 0.05	4.93 ± 0.05

Antibiotic activity (halo [mm])	17.89 ± 1.38 A	11.35 ± 0.62 B	7.95 ± 3.13 B
Hemolytic activity	1 A	0 B	0.4 B
Lipolytic activity	0.93 A	0.07 B	1.00 A
*lrp* mutation	No	Yes	No

a The first three phenotypic characteristics or traits are those used in clustering analysis (Fig. 1B). For other traits, letters indicate significant differences among groups, as determined by a pairwise Wilcoxon test with Holm multiple-test correction. Dye adsorption, hemolytic and lipolytic activities are indicated here as the proportion of variants with high activity, and the significance of differences among groups is then tested by a pairwise comparison of proportions. For each group, we indicate whether or not variants carry a nonsynonymous mutation in *lrp*. G2#31 is one exception to the rule we indicate here, as it clusters with group 2 variants (Fig. 1B) but has no *lrp* mutation.

The remaining 20 isolates were heterogeneous: they were all motile and had large cells, but 15 of these isolates always formed blue colonies on NBTA medium, while 5 did form both blue and red colonies (Fig. 1A). A clustering analysis restricted to these three quantitative phenotypes (motility, cell size, and proportion of red colonies) confirmed that four isolates formed a third distinct group of variants (isolates G3#9, G3#22, G3#48, and G3#27 [Fig. 1B]). We added isolate G3#47 to this group 3, although it clusters with the first group, because it formed red colonies. Group 3 variants repeatedly combined some characteristics of group 1 variants (large cells, high motility, and high level of lipolytic activity) and other characteristics of group 2 variants (low level of antibiotic activity and, most of all, red color colonies on NBTA [Table 1]).

### Spontaneous mutations in *lrp* produce the phenotypic difference between group 1 and group 2.

Defective *lrp* mutants are not motile and have reduced antibiotic and hemolytic activities (20), just like the group 2 isolates of our collection. It is therefore possible that the group 2 variants are *lrp* mutants. To test this hypothesis, we sequenced *lrp* and its promoter region for the 34 isolates of our collection. All group 1 and group 3 isolates had an *lrp* sequence identical to that of the F1 reference genome (26). Conversely, 13 out of the 14 group 2 isolates had one nonsynonymous mutation. Isolate G2#31 (Fig. 1A) was the sole group 2 isolate with no mutation. Interestingly, G2#31 also stands apart as the group 2 isolate with the lowest *in vitro* growth rate (see Additional file 2 at https://figshare.com/s/4cf02d0aa870dd20d5ab). To discard the possibility that there are other mutations that could be responsible for the phenotype, we performed complementation by inserting a functional copy of *lrp* in the chromosome of G2#25, which has a single nucleotide polymorphism (SNP) in *lrp* codon 120. The complemented variant with group 2 phenotypes showed a full restoration of the phenotypes that are typical of group 1 variants (see Additional file 3 at the above URL).

**FIG 2:**
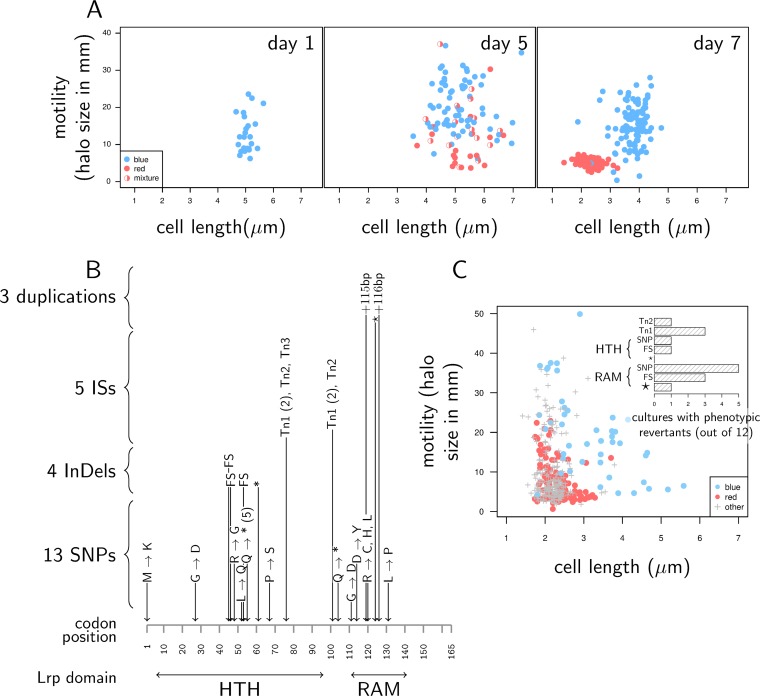
Group 2 variants are *lrp* mutants. (A to C) Cell size and motility measured on clones sampled in 24 independent static cultures of variant G1#23. Data are shown after 1, 5, and 7 days of incubation. Filled circles (blue or red) represent clones producing only one colony color, while half-filled circles represent clones producing mixtures of red and blue colonies when streaked on NBTA. (B) Summary of mutations found in the regulatory gene *lrp*. All the *lrp* mutations we found in group 2 variants were located in the coding sequence. HTH (helix-turn-helix) is the protein domain that contains the Lrp DNA-binding site, while RAM (Regulation of Amino acid Metabolism) is the coregulator response domain of Lrp. FS stands for frameshift, and asterisks indicate nonsense mutations that cause the truncation of the translated protein. For SNPs that cause a substitution, the change in amino acid is given. Tn1, Tn2, and Tn3 are three transposons. Numbers in parentheses give the number of independent replicate cultures where a mutation has been observed (one if not indicated). The mutations we found are grouped here in four categories: three mutations were tandem duplications of a 26- to 116-bp-long fraction of the *lrp* sequence; five mutations were insertion sequences (ISs) with Tn1, Tn2, and Tn3 being three different transposons which belong to the group of IS5 insertion sequences, and share the same two insertion points in *lrp*; four mutations were insertions or deletions of a few bases (indels); 13 mutations were single nucleotide polymorphism (SNPs). (C) Cell size and motility of cells sampled from prolonged culture of eight different group 2 isolates (one sense mutation, one nonsense mutation, and one frameshift in either the HTH domain or RAM coregulator response domain, and two transposon insertions). The colors of the symbols indicate the color of colonies on NBTA medium; gray crosses indicate that the color was neither blue nor red. Eight days after cultures were started, we observed cells that have recovered part of the functions typical of group 1 variants. Many of these reversions, however, are not complete: some revertants have large cells but remain nonmotile; other are motile but have small cells or are still red. (Inset) Number of cultures (out of the 12 we performed for each isolate) where blue or hemolytic colonies were observed. HTH and RAM identify isolates that have a mutation in either of the two main active domains of the Lrp protein. As in Fig. 1C, Tn1 and Tn2 are two distinct insertion sequences, FS stands for frameshift, SNP indicates sense point mutations, and the asterisks indicate nonsense mutations.

To validate that these results were not only restricted to our collection, we initiated 24 independent *in vitro* cultures from the group 1 variant G1#23, which we plated every day on NBTA medium. Beyond being an experimental replicate, this experiment allowed us to monitor the changes of phenotypes over time (at 1, 5, and 7 days). As expected, all clones initially formed blue colonies and were motile (Fig. 2A). After 5 days of incubation, we were able to sample bacteria forming red colonies (Fig. 2A). They did not differ from other isolates in terms of cell size, but they had significantly lower motility and antibiotic activity (by Kruskal-Wallis rank sum test, *P* = 1.41e−04 and *P* = 1.08e−05 for motility and antibiotic activity, respectively, using measurements that were first averaged by culture). After 7 days of incubation (Fig. 2A), bacteria forming red colonies were small nonmotile cells, while those forming blue colonies were large motile cells (Kruskal-Wallis rank sum test, *P* < 2.2e−16 for both cell size and motility averaged by culture). Over the 7 days of culture, the frequency of red variants increased from 0% to about 20%.

We then sequenced *lrp* in clones sampled after 8 days of incubation and found nonsynonymous mutations in 15 out of the 18 sampled red colonies. Red variants sampled at day 5 were also *lrp* mutants, although in a smaller proportion (8 out of 14 sequenced clones). We then averaged the measurements performed on these clones by culture and found that red variants with and without *lrp* mutations had similarly low antibiotic activity (Wilcoxon test, *P* = 0.26) but that red variants with no *lrp* mutation were more motile (Wilcoxon test, *P* = 2.66e−3 and *P* = 1.65e−3 at day 5 and day 8, respectively) and had larger cells than red variants with *lrp* mutations at day 8 (Wilcoxon test, *P* = 1.65e−3). They therefore match our definition of group 3 (Table 1). Furthermore, their frequency decreased from day 5 to day 8, which suggests that group 3 variants appeared earlier and were replaced by group 2 variants. This is confirmed by another replicate experiment, where we found red colonies appearing early to be significantly more motile than those appearing late (see Additional file 4 at https://figshare.com/s/4cf02d0aa870dd20d5ab).

### *lrp* mutations are diverse and not reversible.

Phenotypic variants in *Xenorhabdus* were considered phase variants (15). If this theory is correct, mutations in *lrp* should be the product of a specific molecular mechanism (6). Overall, we identified 25 distinct nonsynonymous mutations in *lrp* (Fig. 2B). Of these 25 mutations, 15 most probably caused profound alterations to the translated protein: five IS*5* insertions and three large duplications totally modified the *lrp* sequence, three indels caused a frameshift, and one indel and three SNPs caused nonsense mutations. The remaining 10 mutations are SNPs that changed a single amino acid in Lrp. The mutations we found in group 2 variants are therefore highly diverse, which makes them unlikely to result from a single specific mutation mechanism.

If group 2 variants were phase variants, *lrp* mutations should also be reversible (6). We thus tested the reversibility of group 2 isolates by monitoring prolonged static LB cultures of eight group 2 isolates with distinct *lrp* mutations. After 8 days of incubation, we observed cells capable of forming blue colonies for most of tested group 2 isolates (Fig. 2C). The sequencing of *lrp* revealed that none of these phenotypic reversions was associated with a genetic reversion of the initial mutation. Accordingly, most of these phenotypic reversions were only partial, with blue colonies being composed of either small or weakly motile cells (Fig. 2C). This confirms that the *lrp* mutations in group 2 isolates are not produced by a phase variation mechanism. Interestingly, the probability of phenotypic reversion differed among the isolates tested (Fig. 2C, inset, glm with binomial error, Chi2 = 31.38, *df *=* *7, *P* = 5.28e−05) which suggests that the way phenotypes are restored in *lrp* mutants depends on the precise nature of the mutation. Finally, we never observed revertants in cultures of the group 3 variants G3#9, G3#22, and G3#48.

### Group 2 and group 3 variants are under positive selection during prolonged *in vitro* culture.

**FIG 3:**
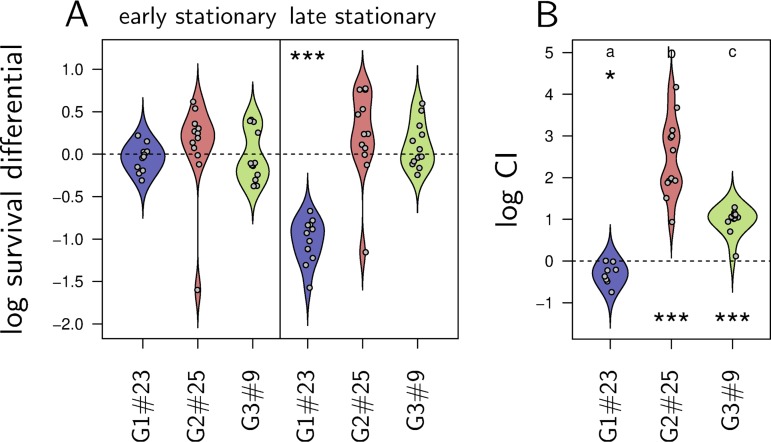
Group 2 and group 3 variants are under positive selection during prolonged *in vitro* culture. (A) Bacterial survival during early and late stationary phase. We estimated the proportion of living bacteria as the ratio between the total number of cells in a LB culture (estimated with a Thoma cell counting chamber) and the number that can form colonies on NBTA. We contrast here the proportion of living bacteria measured in early or late stationary phase to that measured during exponential phase. Asterisks indicate significant deviations from zero, as tested by a Wilcoxon test. Survival does not significantly vary between early stationary phase and exponential phase. During late stationary phase, G1#23 experiences a 10-fold decrease in survival, while G2#25 and G3#9 survival does not vary significantly. (B) Log competitive index (CI) of G1#23, G2#25, and G3#9 inoculated in a F1V1 (non-GFP variant from group 1) culture. For each culture, CI is estimated as the variation in proportion of GFP-labeled variants. Asterisks indicate significant deviation from zero, as tested by a Wilcoxon test. As expected, the group 1 GFP-labeled variant G1#23 shows a marginal decrease in frequency when inoculated in the non GFP-labeled group 1 F1V1. Conversely, G2#25 and G3#9 both increase in frequency.

Variants of *X. nematophila* reach high frequency in prolonged *in vitro* cultures, which suggests they are under strong positive selection. To test this, we first estimated bacterial survival during stationary phase (Fig. 3A). All three variants tested had the same survival during early stationary phase (Wilcoxon test, *P* > 0.07), but G1#23 has a 10-fold-lower survival in late stationary phase compared to early stationary phase (Wilcoxon test, *P* = 5e−4), while G2#25 and G3#9 maintained high survival (Wilcoxon test, *P* > 0.09). Group 2 and group 3 variants therefore seem to resist better than group 1 variants to the stressful conditions of late stationary phase, explaining why group 2 and group 3 variants reach higher densities than group 1 variants in prolonged culture (see Additional file 2 at https://figshare.com/s/4cf02d0aa870dd20d5ab).

We then investigated competitive ability of the same three isolates (Fig. 3B). For this purpose, we inoculated G1#23, G2#25, and G3#9 in a LB culture of F1V1, a non-GFP-labeled group 1 variant. We then quantified the variation of frequency of green fluorescent protein (GFP)-labeled variants (competitive index [CI]): over 5 days of incubation, G1#23 decreased slightly in frequency (Wilcoxon test, *P* = 0.016), while G2#25 and G3#9 both increased in frequency relative to the nonfluorescent competitor (Wilcoxon test, *P* = 4.88e−4 in both cases). G3#9 had an intermediate competitive advantage (Wilcoxon test, *P* = 1e−4) which correlates with the previous observation that group 3 variants had intermediate phenotypes. Altogether, our results suggest that phenotypes of group 2 and group 3 variants have a growth advantage in stationary phase (GASP [25]) and are therefore under positive selection in aged cultures.

### Group 2 and group 3 variants also appear inside insects.

**FIG 4:**
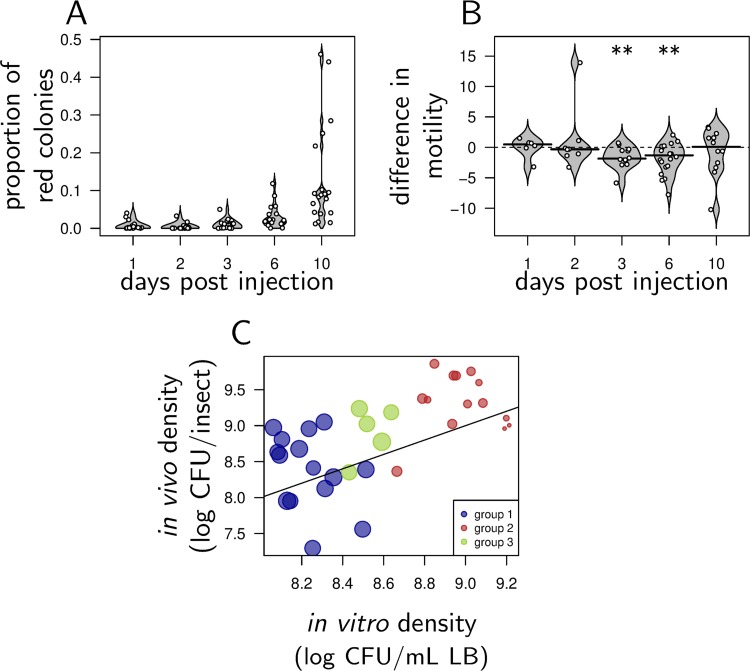
Group 2 and group 3 variants increase in frequency in the insect during infection. (A) Increase in the frequency of red colonies over the 10 days of an *in vivo* experiment. (B) Difference in motility between red and blue cells sampled in insects. Asterisks indicate that the difference in motility was significantly different from zero, as tested by a Wilcoxon test. Compared to blue isolates, red isolates are initially as motile (as expected for group 3 variants) but become less motile at day 3 and 6 (as expected for group 2 variants). (C) Log CFU per insect (5 days after injection; see Materials and Methods) as a function of log CFU per ml in agitated LB culture (after 91 h of incubation; see Materials and Methods). Each symbol on a graph corresponds to one of the 34 isolates of the collection. The color of the symbol indicates the group the variant belongs to, and the symbol size increases with increasing cell size (approximated by log FSC as in Fig. 1A). Bacterial density in insects strongly and positively correlates to density *in vitro* which suggests that traits favored in aged LB cultures are also favored during late infections.

Akhurst, in the first description of *Xenorhabdus* variants (13), mentions that they appear *in vitro* but also in insects during infection. To test this in the case of our particular strain of *X. nematophila*, we monitored the appearance of group 2 and group 3 variants after injecting the group 1 isolate G1#23 in Galleria mellonella. As observed *in vitro*, red variants increased in frequency and reached 10% of the CFU on average 10 days after injection (Fig. 4A). Red colonies were initially slightly (although nonsignificantly) more motile than blue colonies, as expected for group 3 variants, but became significantly less motile at both days 3 and 6, as expected for group 2 variants (Fig. 4B). Lack of difference at day 10 comes mostly from a late increase in motility in red variants (e.g., average motility is 9.73 ± 1.74 mm in red variants at day 3 and increases to 13.7 ± 3.15 mm at day 10). Finally, we found that red variants isolated from infected G. mellonella were *lrp* mutants when they had group 2 phenotypes, but they carried no *lrp* mutation when they had group 3 phenotypes.

The increase in variant frequency in infections (Fig. 4A) suggests that the conditions that favor group 2 and 3 variants *in vitro* may also apply inside insects. To test this, we measured bacterial densities of the 34 isolates of our collection 5 days after injection in G. mellonella larvae. We found that the density *in vivo* positively correlated with the density variants reached *in vitro* (Fig. 4C; Kendall correlation, τ = 0.40, *P* = 0.7e−4): the variants that best performed *in vitro* also reached high densities in insects.

### Group 2 variants are less virulent and least transmitted.

**FIG 5:**
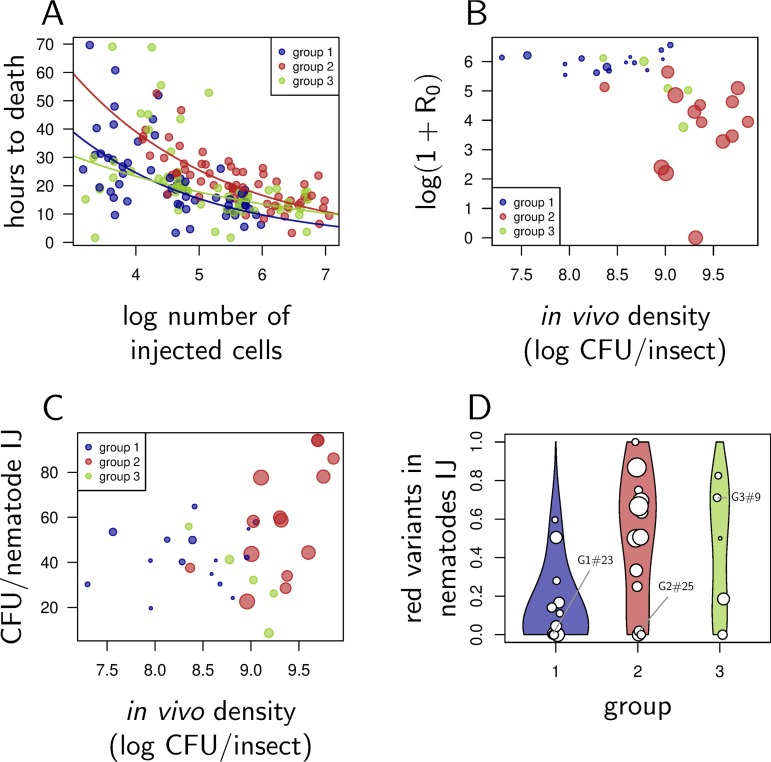
The most competitive group 2 and group 3 variants are the least transmitted. (A) Time to death (in hours after injection) for five isolates of each group, as a function of the log-transformed number of injected cells. Each circle represents the value for a single insect, for which the density of inoculum has been estimated so that the number of injected cells could be computed. Curves correspond to the prediction of a survival regression performed on each group of variants. They illustrate the fact that for a given inoculum size, group 2 variants take longer to kill the insect. (B) *R*_0_, a measure of *Xenorhabdus* transmission, as a function of the log CFU per insect. This measure of bacterial density is shown on the *y* axis of Fig. 4C. *R*_0_ incorporates nematode parasitic success, reproductive success, and survival and the number of bacteria carried on average by a nematode IJ. Each circle shows the value for an individual isolate (total of 34 isolates), and the size of the circle increases with the log number of CFU per ml the isolate reaches on average in LB cultures (shown on the *x* axis of Fig. 4C). (C) Average number of bacteria per nematode IJ as a function of the log CFU per insect. Again, the measure of bacterial density is shown on the *y* axis of Fig. 4C, and the size of a circle increases with the average number of CFU per ml reached in LB cultures. (D) Average proportion of red isolates per nematode IJ as a function of the variant group used to start the infection. The size of circles increases with the number of bacteria carried per IJ. Low values in group 2 and 3 indicate that reversion occurred during infection, and that most IJs carry revertants.

Transmission of *Xenorhabdus* relies in part on their capacity to kill the insect host. We found that all variants retained this capacity, although group 2 variants took slightly more time than others to kill G. mellonella (Fig. 5A and Table 2). We then calculated a proxy of bacterial transmission (*R*_0_) for each of the 34 isolates of the collection. This quantity incorporates the parasitic and reproductive success of nematodes, the number of *Xenorhabdus* bacteria carried per injective juvenile (IJ) and IJ survival during dispersal; it predicts the number of new infections that can be initiated from a single infected insect (11). We found that transmissions were comparable in group 1 and group 3, while group 2 variants had a much lower transmission than the other groups (Fig. 5B, pairwise Wilcoxon test with Holm correction, *P* = 0.14 and *P* = 3.1e−07, respectively). Reduced transmission in group 2 variants was explained by lower parasitic success, reduced fecundity of nematodes, and an increase in the death rate of nematodes (Kruskal-Wallis test, *P* = 1.11e−5, *P* = 3.40e−5, and *P* = 2.85e−4, respectively). For a complete and detailed analysis of the components of *R*_0_ transmission, see Additional file 5 at https://figshare.com/s/4cf02d0aa870dd20d5ab. Isolates that reached the highest *in vivo* (and *in vitro*) loads were the least transmitted (Fig. 5B, Kendall’s rank correlation, τ = −0.43, *P* = 1.946e−4), although they did reassociate with nematodes (Fig. 5C, Kendall’s rank correlation, τ = 0.26, *P* = 0.032). These isolates were not transmitted mostly because they impaired nematode reproduction (Kendall’s rank correlation between *f* and *in vivo* CFU, τ = −0.39, *P* = 8.55e−4).

**TABLE 2:** Analysis of survival data^*a*^

Effect	Chisq	df	*P* value
Log CFU injected	94.92	1	<2.2e−16
Group	17.93	2	1.21e−04
Interaction	4.15	2	0.13

a Time to death has been analyzed using a Cox proportional hazard model. The model includes two random effects that account for variation among replicate experiments and variability among variants within each group. Each simple effect (log CFU injected and group) has been tested, while the other simple effect was kept in the model. Therefore, the significant effect of group cannot be explained by a difference in inoculum size among groups.

As expected, switching occurred during infections: group 1 infections produced some IJs which carry red variants, while the majority of group 2 infections produced some IJs carrying blue variants (Fig. 5D). In contrast to what we observed *in vitro*, we found that group 3 variants did revert in the insect, with group 3 infections producing IJs that carry bacteria forming blue colonies. Overall, our data indicate that the proportion of group 2 and group 3 infections producing IJs carrying blue revertants is significantly higher than the proportion of IJs emitted from group 1 infections that carry red variants (Wilcoxon test, *P* = 1.34e−3).

## DISCUSSION

Xenorhabdus nematophila secondary variants are characterized by a well-known suite of phenotypic traits (inability to adsorb bromothymol blue, reduced motility, reduced antibiotic, hemolytic, lipolytic, and proteolytic activities [12–14]) to which we added in this work their smaller cell size and the fact that they better survive and reach higher densities than group 1 in prolonged culture. Earlier studies have demonstrated that group 2 variants share many of these traits with *lrp* defective mutants, and it has therefore been proposed that Lrp was controlling phenotypic variation in *X. nematophila* (20). Using a cohort of isolates, we demonstrate here that the switch to secondary forms in *Xenorhabdus* is caused by *lrp* mutations. But in contrast to what is expected in phase variation (5), these mutations were of diverse nature, and we never observed complete phenotypic reversions involving the restoration of a functional *lrp* sequence. Overall, our data therefore demonstrate that secondary variants of *X. nematophila* are not phase variants, but instead plain *lrp* mutants. The fact that we observed reversion at rates that vary among *lrp* mutants suggests that reversion is achieved through a variety of compensatory mechanisms, probably involving several Lrp regulated genes that are yet to be identified.

Surprisingly, we never observed synonymous mutations in *lrp*. This indicates that *lrp* is not a mutational hot spot: the *lrp* mutants we have found are probably part of the standing genetic diversity present at a low frequency inside the bacterial population, frequently detected in our experiment because they are under positive selection.

Lrp belongs to a family of global regulators that are known to respond to nutrient availability and regulate cell metabolism in case of food shortage (27, 28). Previous work (18) and our *in vitro* measurements (Additional file 2 at https://figshare.com/s/4cf02d0aa870dd20d5ab) demonstrate that *Xenorhabdus lrp* mutants grow more slowly in rich culture medium, but we found that they survive better and reach 10-fold-higher loads than nonmutants during prolonged stationary phase. Most importantly, *lrp* mutants did outcompete group 1 variants in aged cultures, a phenotype described as growth advantage in stationary phase (GASP [25]). This interpretation is further strengthened by the fact that one of the first GASP mutants identified was an *lrp* mutant of Escherichia coli K-12 (29, 30). We therefore propose that secondary variants in *X. nematophila* are under selection during late infection because they are GASP mutants.

We also documented here the existence of a third class of phenotypic variants, which do not carry *lrp* mutations and share phenotypic characteristics with both group 2 variants (red colonies, reduced antibiotic activity) and group 1 variants (large and motile cells, lipolytic activity). Interestingly, such a combination of traits has been reported in some variants that Cowles et al. (20) considered secondary forms but that might in fact be similar to our group 3 variants (Table 2 in reference 20). These variants outcompete group 1 variants and thus display a GASP phenotype, although weaker than that we measured in group 2 variants. The genetic or epigenetic mechanism responsible for the emergence of this phenotype is yet to be identified.

*Photorhabdus*, the sister genus of *Xenorhabdus*, provides additional evidence that pathogens can produce a variety of forms during infection. *Photorhabdus* produce both secondary variants and M forms, another type of variant which is the only one capable of reassociating with the nematode vector (31). However, to our knowledge, Lrp is not involved in the production of any kind of variant in *Photorhabdus*. Although the two genera have very similar life cycles, *Photorhabdus* therefore seems to produce variants that differ from those described for *Xenorhabdus*.

We showed that *Xenorhabdus* variants of group 2 and 3 better resist the conditions of *in vitro* late stationary phase compared to group 1 variants. We found indications that the combination of traits that make them more competitive under these conditions is also advantageous in the insect: the variants that reach the highest densities in aged LB cultures also reach the highest loads in late infections. This explains why variants of group 2 and 3 repeatedly appear in insects. This also asks the question of their adaptive value in the natural situation where they interact with their nematode vector. In fact, *lrp* mutants are poorly transmitted, which cannot be explained by a deficiency in reassociation with nematodes, as we found that IJs emitted from group 2 infections carried as many bacteria as those from group 1 infections. This observation contradicts the prediction of Cao et al. (22) but supports previous findings by Sicard et al. (19). Low transmission of group 2 comes instead from a sharp decrease in nematode reproduction, which is in agreement with earlier experimental results (20, 22) and corresponds with the well-established detrimental effect of secondary variants in mass production of *S. carpocapsae* (32). We also found that IJs emitted from group 2 infections have lower survival during dispersal compared to group 1 infections, which is probably yet another indication that infections initiated with *lrp* mutants constitute an unfavorable environment for *S. carpocapsae*, which is consistent with the study of Cao et al. (22). These observations may be compared to those of Morran et al. (33) who have shown that strong positive selection of virulence in *X. nematophila* negatively affects its transmission by *S. carpocapsae*. Finally, we found that a high proportion of IJs produced from group 2 or group 3 infections carry group 1 variants. This might be because reversion occurs in insects at a higher rate than in culture or because dispersing nematodes preferentially associate with blue variants when mixed with red ones. In any case, our data suggest that group 2 and group 3 variants are counterselected during the transmission phase of *Xenorhabdus*.

Chapuis et al. (11) have demonstrated that death rate of IJs increases with the number of *X. nematophila* cells they carry. As a result, survival of IJs during dispersal trades off against their capacity to initiate a new infection. Here, we show that variants that reach higher loads in late infections are the least transmitted by nematodes (Fig. 4C). This can be understood as yet another trade-off, distinct from that demonstrated by Chapuis et al. (11). Traits favored during infection because they permit high bacterial loads are disfavored during transmission because they decrease nematode reproduction. In E. coli, *lrp* mutants are thought to be better adapted to late stationary phase in part because they scavenge some of the amino acids they need, instead of producing them (29, 30). In *X. nematophila*, *lrp* also controls the production of exoenzymes which are known to support nematode reproduction (20–24), and this advantage would come at the cost of a reduction in transmission.

**FIG 6:**
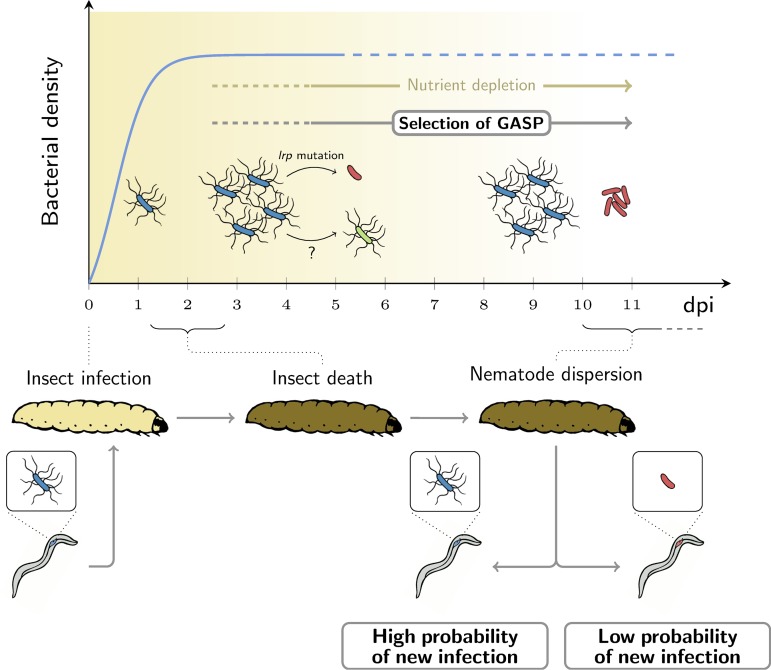
A scenario for selection of GASP variants in long-term batch cultures and over the life cycle of *X. nematophila*. (Top) Bacterial growth over the course of the infection (inspired by Fig. 1 in reference 25). (Bottom) Key steps in *X. nematophila-S. carpocapsae* life cycle over the same time scale shown in the top panel. From 3 days postinfection (dpi), GASP variants (group 2 *lrp* mutants in red and group 3 in green) are detected and rapidly increase in frequency because they better resist the conditions of stationary phase than primary variants (in blue). When nematodes start dispersing around 10 to 15 dpi, the population of *X. nematophila* within the host cadaver may comprise a high proportion of GASP variants. In principle, GASP variants, could therefore contribute to transmission. However, our data suggest that nematodes that carry GASP variants have a lower probability to succeed in infecting new insects.

Here, we have investigated the diversity of phenotypic forms that appear in prolonged cultures of *X. nematophila*. We found that these forms can be classified in several distinct groups, which have in common that they have GASP phenotypes and thus increase in frequency both *in vitro* and during late infections, inside the insect host. We finally demonstrate that the variants that reach their highest loads, both *in vitro* and in insects, are those that are the least transmitted, because they negatively impact the reproduction of their nematode vectors (Fig. 6). Similar situations could probably arise in other pathogens that stay for many generations inside their host (34, 35). GASP mutants, which can impact transmission, may therefore influence the evolution of pathogens that form long-lasting infections.

## MATERIALS AND METHODS

### *X. nematophila* isolates.

Xenorhabdus nematophila isolates were obtained from static cultures of the green fluorescent protein (GFP)-labeled strain F1D3 (19). Samples of 10 independent LB cultures of strain F1D3 were streaked onto NBTA plates (13) after 3, 7, and 13 days of incubation at 28°C. Primary forms form blue colonies on NBTA, while secondary variants form red colonies (12, 14, 15). Each time a red colony was observed, a blue colony from the same petri dish was also sampled. Overall, we obtained 34 distinct isolates (see Additional file 1 at https://figshare.com/s/4cf02d0aa870dd20d5ab) which we stored in 20% glycerol at –80°C.

### *lrp* sequencing.

*X. nematophila* colonies were lysed in sterile Milli-Q water after several freezing-thawing cycles. The *lrp* gene was amplified using the high-performance GoTaq G2 DNA polymerase (Promega, France) with primers lrp-L (5′-CATATTGCGGATTTAGGG-ATTG-3′) and lrp-R (5′-GGGACTGCATAGGCAAGAATAC-3′). PCR products were sequenced by GenoScreen, France, and mutations were determined by comparing aligned *lrp* sequences to that of the *X. nematophila* F1 reference genome (26).

### Measuring colony phenotypes.

For each isolate, we measured four phenotypic traits that differ among *Xenorhabdus* variants. Swimming motility was measured as the diameter of a halo formed by motile bacteria on 0.35% agar culture medium (14, 15). Antibiotic activity was quantified by measuring the diameter of inhibition halos, using Micrococcus luteus as a target strain (36). Extracellular lipolytic activity was assessed by the presence of precipitated material surrounding the colony cultured on Tween 20 agar (36). Hemolytic activity was quantified by the presence of a clearing surrounding bacteria grown on standard sheep blood agar plates (37).

### Measuring cell size.

Cell size was first estimated by flow cytometry analysis in three replicate *in vitro* experiments. In each experiment, exponential-phase cultures of each isolate were fixed with a 0.2% solution (vol/vol) of formaldehyde and analyzed with a FACSCalibur flow cytometer (Becton Dickinson), equipped with an argon air-cooled laser providing 15 mW at 488 nm and the standard filter set-up. Bacterial cells were discriminated from particles by applying a polygonal gate to green (530 ± 15 nm) and red (585 ± 21 nm) log-transformed fluorescence measurements and a rectangular gate on log-transformed side scatter (SSC) and forward scatter (FSC), using tools provided in R Bioconductor (38). We used log-transformed FSC as a proxy for cell size.

In other experiments, when cytometry was not applicable, we sampled colonies, put them on cover slides, and took pictures with an Olympus BX51 microscope with 400× magnification. Images were analyzed using ImageJ (39), and the size of a cell was approximated by the maximum Feret diameter of the cell contour. We measured a minimum of 10 cells per sample and used the average Feret diameter (in microns) as a cell size estimate.

### Measuring bacterial density and survival.

*In vitro* bacterial density during stationary phase was measured on agitated LB cultures incubated at 28°C for 91 h. Five replicate experiments were performed, and appropriate dilutions were streaked on NBTA plates with 50 μg kanamycin per ml to estimate density. *In vivo* bacterial density was estimated by injecting approximatively 2000 cells (i.e., 20 μl of cultures diluted to 1:1,000) in the last instar larvae of the lepidopteran Galleria mellonella as previously described (19). Insect cadavers were homogenized in 100 μl LB after 5 days of incubation at 28°C, and appropriate dilutions were streaked on NBTA to estimate density.

We estimated bacterial survival on three isolates (G1#23, G2#25, and G3#9) for which we ran 12 independent agitated LB cultures at 28°C. Samples were taken from each culture in late exponential phase, early stationary phase, and late stationary phase. For each sample, we estimated the total number of cells using a Thoma cell counting chamber and the number of cultivable cells by plating appropriate dilutions of samples on NBTA plates and counting the number of CFU. We then used the proportion of cultivable cells as a proxy for the proportion of viable cells and studied how it varied from exponential phase to early or late stationary phase.

### Measuring competitive ability.

We measured competitive ability of three fluorescent isolates (G1#23, G2#25, and G3#9) by letting them compete in LB cultures with a nonfluorescent primary variant (F1V1). Twelve independent late-stationary-phase cultures of each of the four isolates were used to inoculate these cultures. Before inoculation, cultures of group 2 and 3 variants were 10-fold diluted so that all groups of variants have similar densities. The two competing variants (125 μl each) were then mixed, incubated for 120 h at 28°C, and plated on NBTA. Pictures of these plates were taken using an Olympus Axiozoom (x7) under fluorescent light (535 nm), so that fluorescent and nonfluorescent colonies could be distinguished. The densities of GFP and non-GFP cells were estimated at the onset of the experiment and after 5 days of incubation. From these estimates, we could compute a competitive index (CI) as the rate of increase in frequency of GFP bacteria over 5 days of incubation.

### Measuring virulence toward insects.

We measured virulence as the time to kill larvae of G. mellonella. This was done for five representative isolates of each group (isolates G1#21, G1#23, G1#42, G1#44, and G1#51 for group 1, isolates G2#25, G2#29, G2#36, G2#39, and G2#40 for group 2, and all members of group 3) and for three injected doses (corresponding to 20 μl of a 1:1,000, 1:100, or 1:10 diluted culture). We conducted two replicate experiments and injected each dose four times. Appropriate dilutions of each injected culture were plated on NBTA to estimate the injected dose. We used the automated procedure described by Parthuisot et al. (40) to measure time of insect death, and we analyzed data with a Cox proportional hazard model, with variation among replicate experiments and variation among isolates within each group considered a Gaussian random effect. This analysis has been performed using the coxme library (41).

### Measuring transmission by Steinernema carpocapsae.

We associated each of the 34 isolates with the nematode vector Steinernema carpocapsae by first infecting G. mellonella with aposymbiotic (i.e., deprived of *Xenorhabdus*) nematodes obtained from *S. carpocapsae* strain SK27 and kept alive for a few months at 8°C in Ringer solution. After 24 h of incubation at 24°C in tubes containing 20 nematodes, insects were injected with the isolates. Insect cadavers were subsequently placed in White traps and incubated at 24°C until new nematode injective juveniles (IJs) dispersed (42). In this experiment, two or three independent cultures of each isolate were performed so that a total of 96 independent inocula were tested against three lots of insects, for a total of 288 tests. Inocula of group 1 isolates were less diluted than others (i.e., 1:100 instead of 1:1,000) so that comparable numbers of bacterial cells were injected for all isolates. We injected 10^2.45 ± 0.57^ cells, as estimated by plating a fraction of the inocula.

For each isolate, we measured the proportion β of infections that yielded new IJs. When all IJs had emerged from insect cadavers, we suspended them in a fixed volume of Ringer solution and counted the number *f* of living IJs in a 20-μl aliquot of each suspension. We then estimated the mortality rate *ν* of newly emerged IJs by placing four groups of five IJs in Ringer solution, incubating them at 28°C in the dark for 17 weeks, and counting surviving IJs (43). We also quantified the number of bacteria *r* carried per IJ by grinding 20 nematodes for each emergence. IJs had previously been cleaned for 10 min in 0.4% bleach and rinsed several times in sterile Ringer solution. They were then placed for 10 min in TissueLyser II (Qiagen) at 30 Hz with three 3-mm glass beads in order to liberate bacterial symbionts. Portions (100 μl) of this suspension were then spread on NBTA plates with kanamycin, and CFU were counted. Finally, following Chapuis et al. (11), we combined these four traits in a single measurement of bacterial transmission, with

$$R_{0}=\text{β}f\;\left[1-\text{exp}\left(-r\right)\right]/\nu$$

The parasitic success β is measured once for each isolate. Conversely, *f*, *ν*, and *r* are measured for each emergence of a given isolate. In order to obtain an *R*_0_ estimate for each isolate, we first averaged *f* [1 − exp(−*r*)]*/*ν over the replicate emergences of this isolate and multiplied this average by β. We present in the Results section analysis performed with nonparametric rank tests for β, *f*, *r*, ν, and *R_0_*. A more-detailed analysis of all components of *R*_0_ is provided in Additional file 5 at https://figshare.com/s/4cf02d0aa870dd20d5ab.

### Variant emergence and reversion *in vitro* and *in vivo*.

We studied the emergence of variants in 24 independent static *in vitro* LB cultures of isolate G1#23 incubated at 28°C. Samples from each culture were streaked on NBTA plates on days 1, 5, and 7. Five red colonies (when possible) and an equivalent number of blue colonies were then taken from each sample, and their cell size and motility (see above) were measured. On some of these colonies, we also measured antibiotic and hemolytic activities (see above). Following this procedure, we could detect group 2 and group 3 variants rapidly after they emerged. A similar procedure was followed to study reversion to the primary form, where we looked for isolates forming blue colonies in prolonged static cultures of group 2 and group 3 variants. We also monitored the emergence of variants after injection of G1#23 in G. mellonella. Twenty insects were homogenized after 1, 2, 3, 6, or 10 days of incubation at 28°C. As for *in vitro* emergence, red and blue colonies were sampled from NBTA plates, and their motility was quantified as described above with two replicate measurements for each colony. In all these experiments, measurements performed on red and blue clones were first averaged by culture before being compared using nonparametric tests.

### Availability of data and materials.

The supplemental files for this article (Additional files 1 to 6) can be found at FigShare (https://figshare.com/s/4cf02d0aa870dd20d5ab). The data sets supporting the conclusions of this article are included within Additional file 6.
